# Combining Use of Captopril and Losartan Attenuates the Progress of *Streptococcus pneumoniae*-Induced Tympanosclerosis through the Suppression of TGF-β1 Expression

**DOI:** 10.1371/journal.pone.0111620

**Published:** 2014-10-31

**Authors:** Wenqing Yan, Jianfeng Li, Renjie Chai, Wentao Guo, Lei Xu, Yuechen Han, Xiaohui Bai, Haibo Wang

**Affiliations:** 1 Department of Otolaryngology Head and Neck Surgery, Provincial Hospital Affiliated to Shandong University, Jinan, P.R. China; 2 Shandong Institute of Otolaryngology, Jinan, P.R. China; 3 Shandong Provincial Key Laboratory of Otology, Jinan, P.R. China; 4 Key Laboratory for Developmental Genes and Human Disease, Ministry of Education, Institute of Life Sciences, Southeast University, Nanjing, P.R. China; University of Missouri-Kansas City, United States of America

## Abstract

**Objectives:**

In this study, using an *Streptococcus pneumoniae*-induced tympanosclerosis (TS) model, we explored the effects of captopril and losartan in the treatment of TS and the possible mechanisms.

**Study Design:**

A prospective experimental animal study.

**Methods:**

We set up the TS models in both guinea pig and wistar rat by inoculation of type-3 *Streptococcus pneumoniae* microorganisms and then treated the animals with the combining use of captopril and losartan. Otomicroscopy was employed to observe the development of TS. Auditory brainstem response was used to test the hearing function of animals. Hematoxylin-eosin and von Kossa staining were performed to determine the morphological changes and calcium depositions. The protein expressions of transforming growth factor β1 (TGF-β1) were assessed by western blot and immunohistochemistry staining, and the mRNA level of TGF-β1 was measured by quantitative reverse transcription- polymerase chain reaction.

**Results:**

The combining use of captopril and losartan attenuated TS responses in terms of a decrease in the TS incidence and the ABR threshold, a reduction of hyalinization and calcification in the middle ear mucosa and the thickness of the mucosa. In addition, the TGF-β1 expression was decreased at both protein and mRNA levels.

**Conclusion:**

Our data indicate, for the first time, that the combining use of captopril and losartan obviously attenuates TS progress through inhibiting the overexpressing of TGF-β1.

## Introduction

Tympanosclerosis (TS) is a common disease that affects the middle ear and tympanic membrane (TM). While the aetiology for TS has not been elucidated as yet, it is widely accepted that TS is a complication secondary to acute and chronic otitis media [1]. The typical pathologic characteristics of TS are acellular hyalin and calcified deposits that accumulate in the TM and the submucosa of middle ear via three consecutive phases, i.e., the initial injury of collagen fibrils, the fibroblastic invasion, and calcifications and occasional ossifications [2]–[4]. However, both the exact factors and mechanisms responsible for this disorder have not been fully elucidated to date. Even so, compelling evidence has revealed that numerous cytokines, which are closely related to the deposition of collagen and calcium phosphate, contribute to pathogenesis of TS [5]–[8].

Among the known cytokines, transforming growth factor β1 (TGF-β1), a sereted regulatory protein, has generated considerable interest in research areas, because it involves in a wide variety of biological activities, such as embryonic development, proliferation and differentiation of cells, immune response and tissue repair [9]–[13]. Interestingly, previous reports demonstrated that the up-regulation of TGF-β1 affects the incidence and prognosis of otitis media, which is thought to be the cause of TS, and also promotes the development of fibrosis in various organs, such as the liver, lung, and kidney, which appears to experience similar pathological alterations in TS despite the different tissues involved [14]–[17], thereby implying a possible linkage between fibrosis and TS.

It is well known that angiotensin-converting enzyme (ACE) and angiotensin II receptor are involved in the pathogenesis of arteriosclerosis, diabetic nephropathy and other ectopic fibrosis diseases [18]–[20]. ACE inhibitor (ACEI) or angiotensin II receptor blocker (ARB), inhibiting the progress of fibrosis at least partly due to the reduction in the TGF-β1 expression [21]–[24], offers a promising pharmacologic strategy for these diseases. The combination therapy of ARB and ACEI is thought to be superior to either agent alone. Captopril or losartan, such a typical drug, which belongs to the category of ACEI or ARB, respectively, is frequently employed in clinic or animal experiments.

With respect to the experiments on TS, of particular interesting is to understand the pathogenetic mechanisms responsible for TS as well to find drugs effective in the prevention of its development. Currently, as little is known about the exact mechanism(s) responsible for TS, there has been still lack of an effective remedy in relation to this disorder. The present study was designed to gain insight into such mechanisms for TS, with special attention given to whether captopril and losartan is effective in the prevention of TS development in animal models of TS.

## Materials and Methods

### Materials

Adult albino male guinea pigs (weight 300–350 g) and male wistar albino rats (weight 200–250 g) were purchased from Animal Centre of Shandong University, P.R. China. Prior to the experiments, otomicroscopic examination was conducted to ensure the animals without external or middle ear infection. The ones that had external or middle ear infection, tympanic membrane adhesions, or perforations before surgery were excluded. All experimental animals were kept at standard laboratory conditions with normal feeding.

Use of animals for these experiments was approved by the Ethics Committee of the Provincial Hospital Affiliated with Shandong University. The animal care and experimental protocol were approved by The Animal Care Committee of Shandong University, P.R. China (NO. ECAESDUSM 20123011).

The Type-3 *Streptococcus pneumoniae* was used as a pathogen (ATCC Manassas VA, USA). In this study, primary antibodies used for western blot included rabbit anti-TGF-β1 polyclonal antibody (ZSGB, Beijing, China), and anti-β-actin antibody (Santa Cruz Biotechnology, CA, USA). BCA protein assay kit was a product from Shenergy Biocolor Bioscience & Technology Company (Shanghai, China). The ECL detection reagents used for western blot detection were from Santa Cruz Bio-technology (Santa Cruz Biotechnology, CA, USA). cDNA Synthesis Kit (TaKaRa Bio, Siga, Japan) was used for reverse transcription. TGF-β1 primers used for quantitative reverse transcription- polymerase chain reaction (qRT-PCR) were designed and synthesized by Beijing Genomics institution (Beijing, China). Captopril and losartan were purchased form Changzhou Pharmaceutical Factory, Changzhou, P.R. China and Chengdu Hengrui Pharmaceutical, Chengdu, P.R. China, respectively.

### Experimental Design and Operating Procedure

60 healthy adult male albino guinea pigs and 60 healthy adult male rats were utilized in this study. Twenty guinea pigs were chosen to serve as control without treatment. The other forty guinea pigs were inoculated type-3 *Streptococcus pneumoniae* microorganisms into the right ears, then were further divided into two subgroups on the basis of drug intervention or not, with twenty subjects in each group respectively. 60 wistar rats were divided into three groups in the same way. The animals were anesthetized intraperitoneally with 10% chloral hydrate under sterile conditions, and then 0.05 ml of a suspension containing 3×10^8^ CFU of type-3 *Streptococcus pneumoniae* was inoculated by myringotomy into the right ear of the guinea pig, while 0.02 ml into the right ear of the rat, to induce acute otitis media and TS. The left ears of animals were untreated. In captopril and losartan treated groups, immediately after the myringotomy, 1 ml captopril and losartan mixture (captopril, 5 mg/ml; losartan, 5 mg/ml) was intraperitoneal injected into the twenty guinea pigs and the twenty rats. The same procedure was repeated for 21 times with one day intervals. The middle ears were observed by otomicroscope to examine the development of acute otitis media and TS every week. TS was identified as white plaques on the TM, and mild and marked opacification were also accepted as TS in the TS group. One subject was euthanized at the end of the first week to examine early histological changes, and the remaining animals were followed up until the end of week 6. In this process, two guinea pigs of the TS group died.

### Evaluation of auditory function in animal models

Auditory brainstem response (ABR) was used to assess hearing loss in response to various stimuli. Anesthesia was induced using 10% chloral hydrate, guinea pigs: 3 µl/g and rats: 4 µl/g. ABR responses were elicited by click stimuli and delivered from the ‘Smart EP’ auditory evoked potential diagnostic system (Intelligent Hearing Systems, Miami, FL, USA) through a tube inserted into the external auditory canal. Stimuli was presented from 80 dB peak equivalent SPL down to 5 dB in 5 dB decrements. Subdermal platinum-coated tungsten needle electrodes were used to record evoked potential. The positive recording electrode was placed at the vertex, while the reference electrode was placed below the ear pinna ipsilateral to the speaker, and the ground electrode was placed on the back. The response to click stimuli was averaged 1024 times, respectively. For analysis, ABR threshold was considered the lowest intensity at which a distinct portion of the biological waveform remained.

### Tissue Preparation

After undergoing the ABR, these animals were placed in a stereotaxic frame and removed the temporal bones. The middle ear mucosa was microscopically separated, and some tissue samples were quickly frozen at −80°C for western blot analysis and qRT-PCR, others were fixed in 4% paraformaldehyde-phosphate buffered saline for 24 h, and then dehydrated with gradually increasing concentrations of ethanol. The tissue samples were embedded in paraffin and then a microtome (Leica 2125 rotary microtome, Wetzlar, Germany) was used to obtain 4 µm thick sections. These prepared slices were used for hematoxylin-eosin, von Kossa, and immunohistochemistry (IHC) staining.

### Hematoxylin-eosin staining for morphological observation

The paraffin sections were baked at high temperature of 65°C for 2 h, followed by xylene dewaxing, and alcohol rinsing. The sections were stained with hematoxylin for 5 min, followed by rinsing in several times with distilled water, and then stained with eosin for 2 min. Finally, the slides were mounted with neutral gum after dehydration through graded alcohol and transparence in xylene. Changes of basic morphology of the middle ear mucosa were observed under the light-microscopy (Olympus BX51, Japan).

### von Kossa staining for calcified deposits

Briefly, sections were incubated with 1% silver nitrate solution, placed under ultraviolet light for 40 min, under a 100-watt incandescent lamp. These slides were rinsed in several changes of distilled water, and then removed unreacted silver with 5% sodium thiosulfate for 5 min. The slides were washed with distilled water, counterstained with nuclear fast red for 5 min, dehydrated through graded alcohol, made transparent with xylene, and mounted with neutral gum. Finally, deposits of calcium were visualized under the light microscope.

### IHC staining for TGF-β1

The paraffin-embedded specimens were deparaffinized and rehydrated. The sections were treated with 3% hydrogen peroxide for 15 min at 37°C to inactivate endogenous peroxidase, and non-specific binding was blocked by treatment with the blocking reagent. The antigen was retrieved at 95°C for 20 min by placing the slides in 0.01 M sodium citrate buffer (pH 6.0). The slides were incubated with the normal goat serum at 37°C for 30 min, and then with the rabbit anti-TGF-β1 polyclonal antibody (1∶100) to each section at 4°C overnight. Subsequently, the slides were incubated at 37°C for 30 min with biotinylated secondary antibody, and then incubated with streptavidin-peroxidase complex at 37°C for 30 min. The appropriate HRP-conjugated goat anti-rabbit IgG antibody (1∶100) was applied for 1 h at room temperature. Phosphate buffered saline instead of rabbit anti-TGF-β1 polyclonal antibody was added for the negative control.

### Protein extraction and western blot analysis

Total proteins were extracted using radio-immune precipitation buffer-protein lysis buffer according to the protocols. The protein content of the samples was measured using the BCA protein assay kit. Then, 35 µg of each protein sample was denatured and separated by 10% sodium dodecyl sulfate-polyacrylamide gel electrophoresis and transferred onto nitrocellulose membranes. The nitrocellulose membranes were blocked for 1 h at room temperature in 5% skimmed dried milk. Then the blocked membranes were incubated for 2 h at room temperature with rabbit polyclonal anti-TGF-β1 (1∶200) or β-actin (1∶2000) antibodies, followed by anti-rabbit IgG-conjugated horseradish peroxidase, and anti-mouse IgG-conjugated horseradish peroxidase at a 1∶2000 dilution for 1 h at room temperature. Finally, protein signals were detected by chemiluminescence with ECL detection reagents and visualized after exposure to X-ray film. The relative optical density ratio was calculated with the Image J software by comparison to β-actin.

### RNA extraction and qRT-PCR

Total RNA from the frozen samples was extracted according to the manufacturer’s protocol using TRIzol Reagent (Invitrogen, Garlsbad, CA, USA). The total RNA (1 µg) was reverse-transcribed to cDNA using random hexamers and superscript reverse transcriptase. The expression of TGF-β1 was examined by qRT-PCR using SYBR green Master Mix kit and Eppendorf AG 22331 Hamburg machine (Germany). The TGF-β1 primers used were: (Forward) 5′- *ACC TGA ACC CGT GTT GCT CT-*3′ and (Reverse) 5′- *CTA AGG CGA AAG CCC TCA AT*-3′. The β-actin primers used were: (Forward) 5′- *GTG GGG CGC CCC AGG CAC CA*-3′ and (Reverse) 5′- *CTC CTT AAT GTC ACG CAC GAT TT*-3′. 40 cycles were performed with the following PCR conditions: pre-denaturation at 95°C for 4 min, denaturation at 95°C for 30 s, annealing at 60°C for 45 s, extension at 72°C for 1 min, and final extension at 72°C for 10 min. The level of TGF-β1 expression in each sample was normalized to the respective expression level. The specificity of each PCR reaction was confirmed by melting curve analyses.

### Statistical Analysis

The statistical analyses were performed with SPSS version 17.0. One-way analysis of variance was applied to analyze data, and *p*<0.05 was accepted as significant.

## Results

### Combining use of captopril and losartan reduced the incidence of TS

No effusion of the middle ear was otomicroscopically observed in untreated animals and the left ears of animals in all experimental groups. Conversely, acute otitis media was evident and effusion was observed in the right middle ears of all animals in TS group at week 1, but no sclerosis was noticed. Slight sclerotic changes appeared as a few white deposits around the umbo were otomicroscopically observed in 42% of the guinea pig samples at the second week. However, with the treatment of captopril and losartan, typical pathological features of TS were observed only in 26% of the guinea pigs sample. As the extension of time, the sclerotic deposits became more extensive and were widespread rather than patchy in the TS group. At the sixth week, 100% of the animals appeared calcification in TMs, whereas, with the administration of captopril and losartan, only 53% of guinea pigs had calcification in TMs. Similar probability occurred in the rat group. At the end of the sixth week, 100% of the rats without treatment presented with apparent TS, which was significantly higher than that (63%) in rats with the treatment of captopril and losartan. The detailed numbers of tympanosclerotic ears in all groups were otomicroscopically detected as shown in Table 1 and 2.

**Table 1:** Otomicroscopic Evidence of TS on Weekly Examination (guinea pigs).

	Week					
	1	2	3	4	5	6
#TS group (%)	0	8 (42%)	14 (82%)	17 (100%)	17 (100%)	17 (100%)
*Captopril and losartantreated group (%)	0	5 (26%)	7 (37%)	9 (47%)	10 (53%)	10 (53%)

# n = 17,

*n = 19.

**Table 2:** Otomicroscopic Evidence of TS on Weekly Examination (wistar rats).

	Week					
	1	2	3	4	5	6
*TS group (%)	0	11 (58%)	13 (68%)	16 (84%)	19 (100%)	19 (100%)
*Captopril and losartantreated group (%)	0	6 (32%)	8 (42%)	9 (47%)	12 (63%)	12 (63%)

*n = 19.

### The intervention with captopril and losartan partially recovered hearing function

**Figure 1:**
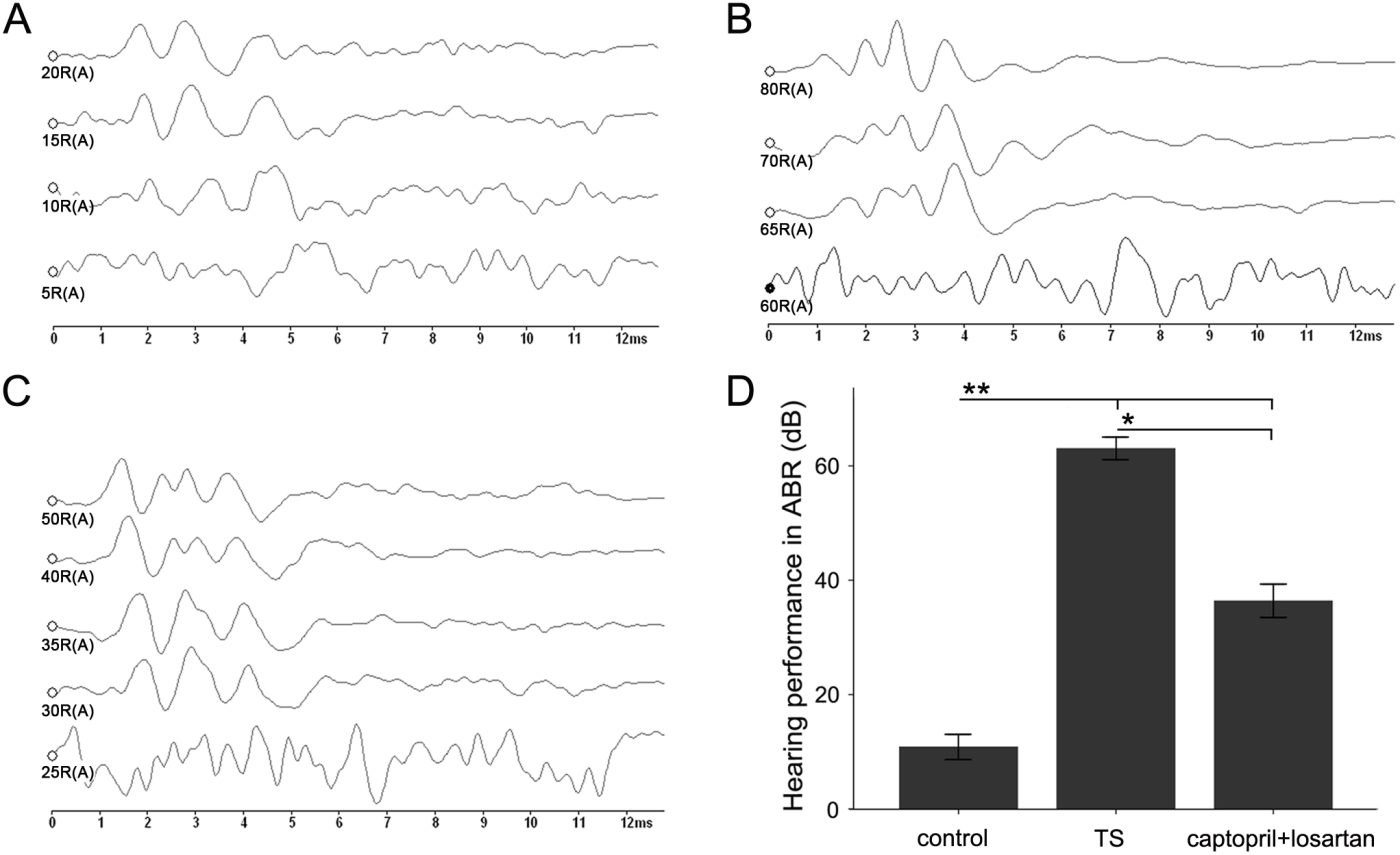
The effect of captopril and losartan on the hearing ability in guinea pig models of TS. The X-axis represents time in milliseconds and Y-axis represents amplitude of the action potentials in microvolts. Each waveform, at a declining dB level to examine response, is stacked onto the same graph. (A) ABR threshold from the control group recorded at week 6. This panel displays a much stronger response to the stimulus and clear waveforms at 10 dB. (B) In TS group, no strong response is shown even at 60 dB. (C) Obvious response can be seen at 30 dB in a guinea pig of the captopril and losartan treated group. The graph in (D) shows ABR thresthold amplitudes in guinea pigs of three different groups. Each bar represents the mean ± standard error of the mean (SEM), n = 19 (the control group), n = 17 (the TS group), n = 19 (the captopril and losartan treated group). *p<0.05, **p<0.01 as conducted.

**Figure 2:**
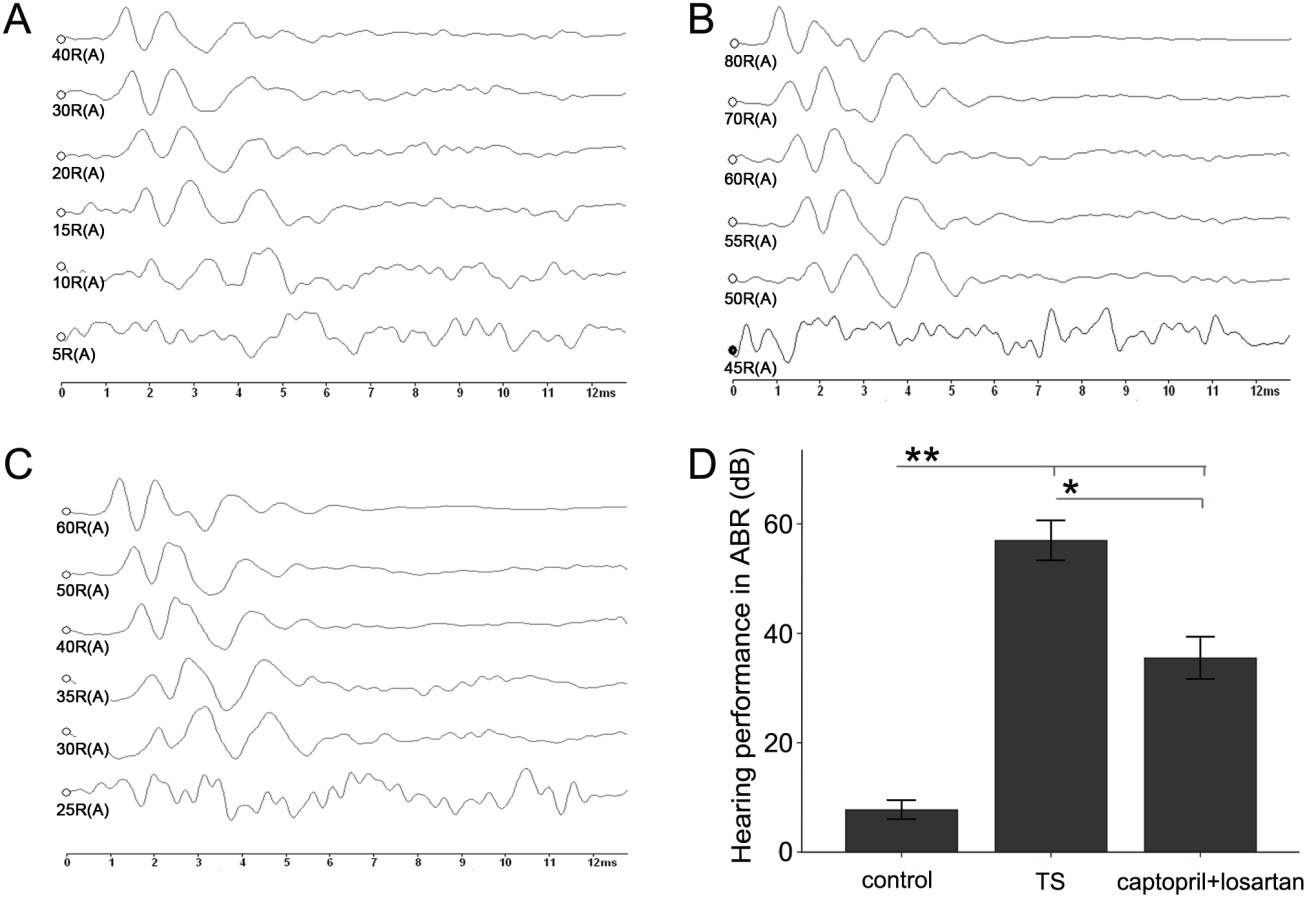
The role of captopril and losartan on auditory function in rat models. (A) In control group, normal hearing threshold can be seen at 10 dB. (B) The hearing threshold is evidently increased in TS group with strong response at 60 dB. (C) After captopril and losartan treated, obvious improvement of hearing threshold is displayed, as clear waveforms shown at 35 dB. (D) This graph shows ABR thresthold amplitudes in rats of three different groups. Each bar represents the mean ± SEM, n = 19 (each of the three groups). *p<0.05, **p<0.01 as conducted.

ABR was performed at the end of week 6 to record the effects of acoustic trauma (Figure 1, 2). This allowed us to determine the difference of hearing function among the three groups. ABR threshold with click was significantly increased in the TS group (guinea pigs, 62.37±1.06 dB and rats, 57±1.83 dB), but was unaffected in control group (guinea pigs, 10.25±1.17 dB and rats, 7.75±0.85 dB), *p*<0.01. This indicated that in TS group, almost all animals showed severe hearing loss after inoculation of the Type-3 *Streptococcus pneumonia* microorganisms into the tympanic cavity. With the captopril and losartan treatment, ABR threshold significantly decreased (guinea pigs, 36.39±1.44 dB and rats, 35.5±1.92 dB) compared with that in TS group without treatment, suggesting that the hearing function partially recovered at week 6 with the captopril and losartan treatment (*p*<0.05). However, compared with that in control, the ABR threshold of animals in captopril and losartan treated group still increased (*p*<0.01), indicating that the recovery of the hearing function was limited. Both the guinea pigs and rats exhibited the similar tendency towards the changes in ABR threshold.

### The combined application of captopril and losartan alleviated the progress of TS as evidenced by histological improvement

**Figure 3:**
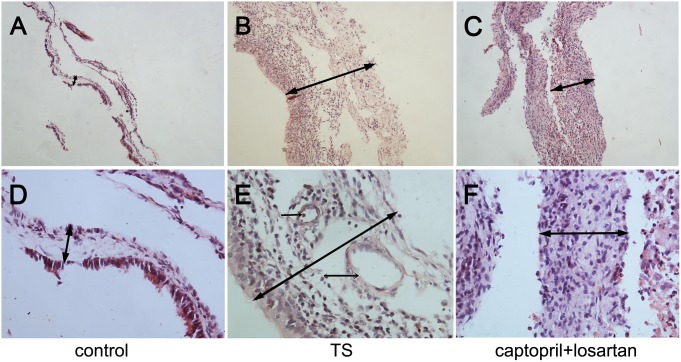
Morphological changes of middle ear mucosa in guinea pigs. Hematoxylin and eosin, original magnification, A, B, C: ×100 and D, E, F: ×400. Normal morphological structure of middle ear mucosa is shown in (A, D). (B, E): In TS group, significantly increased thickness, inflammatory cell infiltration, fibroblast proliferation and vascularization are illuminated. (C, F): In captopril and losartan treated group, the stroma is much thinner and vascularization is reduced than that in the TS group. Double-headed arrow: membrane thickness; arrow: vascularization.

**Figure 4:**
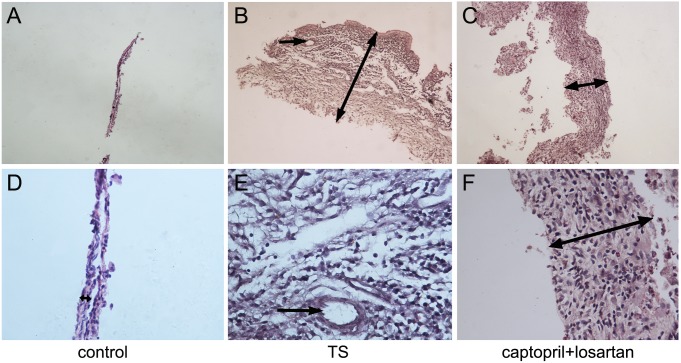
Morphological changes of middle ear mucosa in rats. Hematoxylin and eosin, original magnification, A, B, C: ×100 and D, E, F: ×400 (A, D). Normal morphological structure is displayed in control group. (B, E) In TS group, inflammatory cell infiltration, fibroblast proliferation and vascularization can be seen, as well as significantly increased membrane thickness. (C, F) The two panels show that, in captopril and losartan treated group, not only inflammatory cells, neovascularization and collagen fibers are decreased, but also the stroma is much thinner than that of the TS group. Double-headed arrow: membrane thickness; arrow: vascularization.

Figure 3A & D and Figure 4A & D showed the normal morphological structure of the middle ear mucosa in normal guinea pigs and rats, respectively. No obvious change was observed in the left middle ear of all six groups. In the middle ear mucosa of animals in TS group at week 6, the thickness of membrane and inflammatory infiltration obviously appeared, as shown in Figure 3B & E and Figure 4B & E. In comparison to the middle ear mucosa of TS animals, the thickness of membrane and inflammatory infiltration were markedly decreased in the middle ear mucosa of the animals in captopril and losartan treated group at week 6. Moreover, in captopril and losartan treated group, inflammatory cells and collagen fibers were significantly decreased compared with those in TS group without treatment. Neovascularization was rarer and stroma cell layer was thinner than that in the TS group, as showed in Figure 3C & F and Figure 4C & F.

### Captopril and losartan treatment reduced the calcified deposits

**Figure 5:**
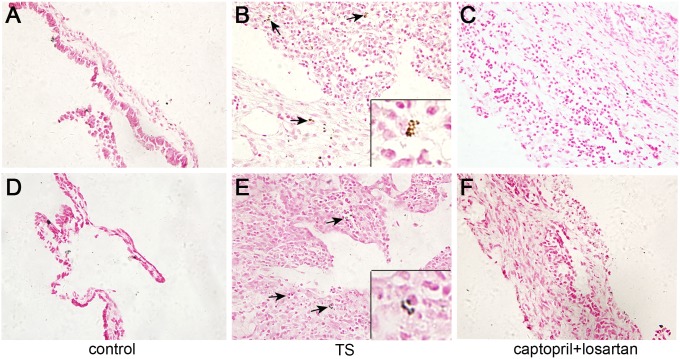
Calcium depositions in the middle ear mucosa. Original magnification, ×400. No obvious calcium deposition is shown in the normal middle ear mucosa of guinea pigs (A) and rats (D). Calcium depositions are appeared as brown yellow granules in TS middle ear mucosa of guinea pigs (B) and rats (E). After captopril and losartan treated, calcium deposition is selodom displayed in guinea pigs (C) and in rats (F). Arrow: calcium deposition.

von Kossa staining was performed to evaluate the calcified deposits, which shown as brown-yellow granules on histological sections. No sclerosis was visible in the middle ear mucosa of the control animals (Figure 5A & D). Slight calcium deposition was observed in the middle ear mucosa of animals in captopril and losartan treated groups (Figure 5C & F), whereas marked calcium crystal accumulation was shown in middle ear mucosa of animals in TS groups and located inside of the cells and interstitial tissue (Figure 5B & E). Similar histologic findings were revealed in guinea pigs and rats.

### Combining captopril with losartan using suppressed the expression of TGF-β1

**Figure 6:**
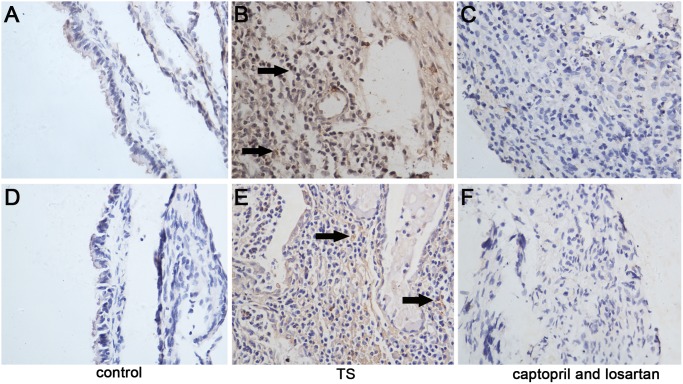
IHC determination of TGF-β1 in the middle ear mucosa. Original magnification, ×400. Few positive cells are observed in normal middle ear mucosa of the guinea pigs (A) and rats (D). Only some positive cells are seen in epithelial cells. In the TS group of the guinea pigs (B) and the rats (E), numerous positive cells are displayed in fibroblasts and inflammatory cells, and mainly locate in cytoplasm of fibroblasts and inflammatory cells. Only few positive cells could be seen in captopril and losartan treated group of guinea pigs (C) and rats (F). Arrow: positive cells.

Evaluation of TGF-β1 protein with IHC staining revealed that few TGF-β1 positive cells were observed in normal middle ear mucosa of guinea pigs, and it was observed only in a small population of epithelial cells (Figure 6A). As shown in Figure 6B, TGF-β1 positive signs were widely distributed in middle ear mucosa of the TS animals at week 6, and it was located in the cytoplasm of fibroblast cells and inflammatory cells. Compared to animals in the TS group, only a few TGF-β1 positive cells were observed in captopril and losartan treated guinea pigs (Figure 6C). Similar to guinea pig, IHC staining in normal middle ear mucosa of rat models revealed that the expression of TGF-β1 was almost undetectable (Figure 6D). TGF-β1 positive cells were obviously increased in the middle ear mucosa of TS animals at week 6, as shown in Figure 6E. Also, only a few TGF-β1 positive cells could be observed in captopril and losartan treated group (Figure 6F).

**Figure 7:**
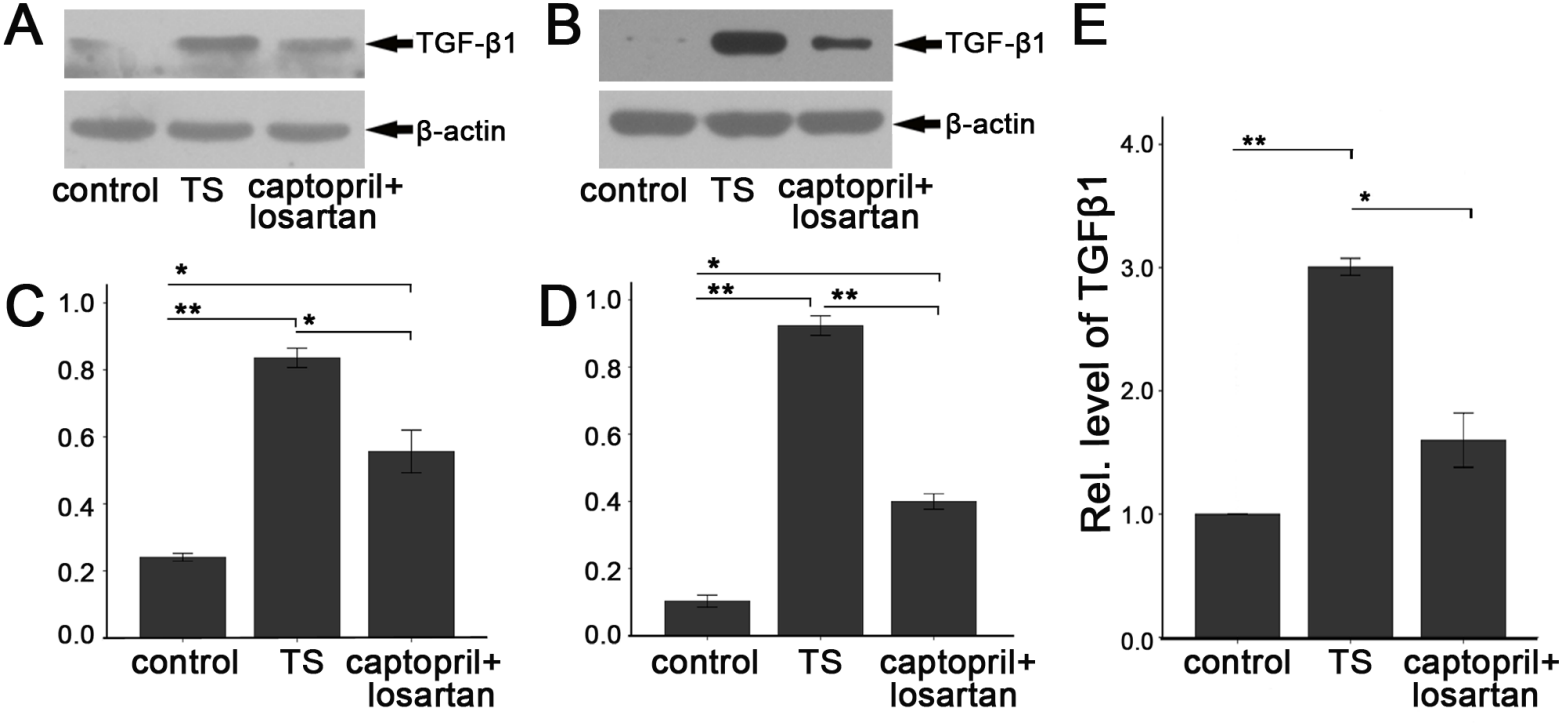
Westeron blot and qRT-PCR detetion of TGF-β1 in the middle ear mucosa. Expression changes of TGF-β1 in protein level are obviously observed in all groups of guinea pig (A) and rat (B). (C) In guinea pig group, statistic analysis reveals that TGF-β1 is increased in TS group, decreased in captopril and losartan treated group but still higher than that of the control group. The same tendency also can be seen in rats group (D). (E) Expression of TGF-β1 in RNA level increases in TS group, and decreases in captopril and losartan treated group at the end of week 6. Each bar represents the mean percent ± SEM, n = 19 (each of the three groups). *p<0.05, **p<0.01 as conducted.

Next, we investigated the protein expression of TGF-β1 in the middle ear mucosa of guinea pig and rat using western blot. As shown in Figure 7A & B, the expression of TGF-β1 was almost undetectable in normal middle ear mucosa of the control groups. However, the expression was apparently upregulated in TS groups at week 6 compared with that in the control groups (*p*<0.01). In addition, the expression of TGF-β1 was significantly decreased in the captopril and losartan treated groups (guinea pig: *p*<0.05, rat: *p*<0.01), but still higher than that in the control groups (*p*<0.05). Similar to the protein expression of TGF-β1, the mRNA expression of TGF-β1 was apparently increased in the middle ear mucosa of animals in TS group compared with the normal middle ear mucosa (*p*<0.01), and significantly decreased in captopril and losartan treated group compared with the TS group at the end of week 6, *p*<0.01 (Figure 7E).

## Discussion

The present study represents the natural extension of our preliminary studies on TS [8]. In this work, we found that the inoculation of type-3 *Spneumoniae pneumonia* microorganisms in the right ears of animals in TS group led to the appearance of hyalinization and calcification in the lamina propria of the middle ear mucous at week 6. On the contrary, neither histologic change nor calcification occurred at any time intervals in the control groups or the untreated left ears. These occurrences of histologic alterations in the TS groups are in accordance with the typical pathological manifestations of TS, indicating that the animal model of TS was successfully set up using puncture and infection and that both guinea pig and rat are species suitable as an animal model of TS, thereby laying a solid foundation for the subsequent experimentations.

It has been documented that ACEI and ARB are effective in the treatment of arteriosclerosis, diabetic nephropathy and other ectopic calciphylaxis diseases through the improvement of vascular remodeling and fibrosis [21]–[24]. While, TS is characterized by the appearance of revasculariation, fibroblast proliferation, and calcium deposit accumulation [1]–[4], [25], which resemble the predominant pathogenic features of the aforementioned disorders. Thus, it is conceivable whether captopril and losartan, are useful in the prevention of TS. In this research, at the end of the first week, no significant effect was observed between the TS group and the captopril and losartan treated group in either guinea pig or rat model. As TS is an end-stage process of continued inflammation, the ears may have not shown the sclerotic signs at this early time point. However, the number of TMs with myringosclerotic plaques kept significantly increasing during the following weeks after the *Streptococcus pneumonia* injection. At the end of week 6, the number of TS formation was much less in captopril and losartan treated group, whereas, TS formation was observed in all animals in the untreated group. These findings suggest that the combined application of captopril and losartan effectively reduced the incidence of TS formation in a time-dependent manner. Moreover, in this work, ABR threshold was significantly elevated in TS groups, which indicated that animals in TS groups showed severe hearing loss after inoculation of the Type-3 *Streptococcus pneumonia* into the tympanic cavity. Conversely, in captopril and losartan treatment group, ABR threshold was significantly decreased, suggesting that, with the intervention of captopril and losartan, the hearing function of animals partially recovered at week 6 compared with TS group, further confirming the effectiveness of the two drugs in TS model.

In addition to the decrease in both TS occurrence and ABR threshold in response to captopril and losartan, histological changes of the middle ear mucosa were also examined in this work. In TS group animals, the thickness of the membrane, inflammatory infiltration, and hyaline degeneration markedly appeared in the middle ear mucosa at week 6. Meanwhile, vascularization and junctional degeneration were also seen in the middle ear mucosa at week 6. In comparison to the middle ear mucosa of TS animals, the thickness of membrane and inflammatory infiltration were markedly decreased in the middle ear mucosa of animals in the captopril and losartan treated group at week 6. Moreover, inflammatory cells and collagen fibers were decreased. These findings provide the histopathologic bases underpinning the inhibitory effects of captopril and losartan on the development of *Streptococcus pneumonia-*induced TS in the middle ear mucosa.

Previous studies on middle ear sclerosis have found that the expression of TGF-β1 was significantly increased in both of the patients [26] with otitis media and the rat model of acute otitis media [27], which indicates that TGF-β1 plays an important role in the development and outcome of otitis media, the most possible cause of TS. Recently, we found that the expression of TGF-β1 in the middle ear mucosa was significantly increased in TS animals and the extent of TGF-β1 expression was positively correlated with the duration of TS [8]. In the present study, we detected that in captopril and losartan treated animals, the protein and RNA expressions of TGF-β1 were significantly decreased in the middle ear mucosa. These findings further confirm that high activity of TGF-β1 may contribute to the pathogenesis of TS and suggest that captopril and losartan exert their preventive effects on the *Streptococcus pneumonia-*induced TS via down-regulation the expression of TGF-β1, which may be one possible explanation for the histological melioration of the middle ear mucosa and the partly recovery of hearing function.

## Conclusion

In conclusion, we demonstrated, for the first time, that the combining use of captopril and losartan obviously attenuates the *Streptococcus pneumonia*-induced TS progress, probably via inhibiting the over-expression of TGF-β1. Our findings may open up possibilities that intervention of captopril and losartan may be used as an alternative strategy for prevention of TS.
